# A CONSULTANT PHARMACIST INTERVENTION TO DEPRESCRIBE OPIOIDS AND BENZODIAZEPINES FOR OLDER ADULTS IN PRIMARY CARE

**DOI:** 10.1093/geroni/igad104.2269

**Published:** 2023-12-21

**Authors:** Joshua Niznik, Jan Busby-Whitehead, Lori Armistead, Benjamin Urick, Liang Zhao, Stefanie Ferreri

**Affiliations:** University of North Carolina at Chapel Hill, School of Medicine, Chapel Hill, North Carolina, United States; UNC Chapel Hill, Chapel Hill, North Carolina, United States; UNC Chapel Hill Eshelman School of Pharmacy, Chapel Hill, North Carolina, United States; Prime Therapeutics, Eagan, Minnesota, United States; UNC Health, Chapel Hill, North Carolina, United States; UNC Chapel Hill Eshelman School of Pharmacy, Chapel Hill, North Carolina, United States

## Abstract

Long-term use of opioids and benzodiazepines (BZDs) by older adults may cause harm. We evaluated the impact of a targeted consultant pharmacist opioid and BZD deprescribing intervention for older adults. We conducted a pragmatic study in which fifteen primary care clinics were randomized to receive standard care (n=7) or a consultant pharmacist service (n=8) to provide targeted medication review and recommendations for deprescribing opioids and BZDs. The primary outcome was opioid and BZD prescribing in the following year, using average daily morphine milligram equivalents (MMEs) and diazepam milligram equivalents (DMEs). We used generalized linear models to evaluate the association of the intervention with average daily MMEs and DMEs, adjusted for medication exposures in the prior year and demographics. Incident falls were examined as a secondary outcome. We included 961 opioid and 1107 BZD users, with 15.6% and 13.6% being prescribed both medications, respectively. Average daily MMEs and DMEs prescribed at baseline were 23.6 and 7.67. Deprescribing recommendations were made for 71% of opioid and BZD users seen in intervention clinics. We observed non-statistically significant reductions in average daily MMEs (Intervention: -2.42 MMEs, Control: -1.02 MMEs, p=0.71) and DMEs (Intervention: -1.0 DMEs, Control: -0.1 DMEs, p=0.06). There was no significant reduction in incident falls. A consultant pharmacist-driven intervention is a feasible approach to deliver targeted deprescribing recommendations, but did not significantly impact medication use or falls. Future studies should explore whether multimodal interventions would be more effective in achieving deprescribing and reducing medication-related harms.

